# Integrated transcriptomic and immune profiling reveals crucial molecular pathways and hub genes associated with postoperative delirium in elderly patients

**DOI:** 10.3389/fmed.2025.1580355

**Published:** 2025-05-09

**Authors:** Zi-han Gou, Nan Su, Xiao-chuan Li, Da-peng Ren, Shan-shan Ren, Lin Wang, Yao Wang

**Affiliations:** ^1^Department of Anesthesiology, The People’s Hospital of kaizhou District Chongqing, Chongqing, China; ^2^Inner Mongolia People’s Hospital Department of Surgical Anesthesia, Inner Mongolia, China; ^3^Department of Orthopaedic, Chongqing Sanbo Changan Hospital, Chongqing, China; ^4^Department of Anesthesiology, The Thirteenth People’s Hospital of Chongqing, Chongqing, China

**Keywords:** postoperative delirium, elderly, cell death, immune microenvironment, WGCNA, PPI

## Abstract

**Background:**

Postoperative delirium (POD) manifests as severe mental disorientation, often experienced by elderly patients undergoing surgery, significantly hindering recovery and deteriorating the quality of life. Despite numerous clinical studies, the molecular mechanisms behind POD in elderly patients are still not well understood, requiring further investigation to identify potential biomarkers and therapeutic targets.

**Methods:**

This study amalgamates Gene Set Variation Analysis (GSVA), Weighted Gene Co-expression Network Analysis (WGCNA), differential expression analysis, and immune infiltration assessments to identify molecular pathways and hub genes linked to the initiation of POD in the elderly. Gene expression data were sourced from the GSE163943 dataset in the Gene Expression Omnibus (GEO) database. A total of 18,894 protein-coding genes were extracted for analysis.

**Results:**

We constructed a gene co-expression network using WGCNA and performed GSVA to investigate the link between POD and different types of cell death. The results indicated that POD is positively associated with pyroptosis and parthanatos, while negatively correlated with oxidative stress and disulfidptosis. Differential expression analysis revealed 145 differentially expressed genes (DEGs), including 83 downregulated and 62 upregulated genes. Analysis of functional enrichment revealed that DEGs were enriched in activities like neuron projection development, axonogenesis, and synapse organization, with KEGG pathway analysis identifying neuroactive ligand-receptor interaction and neurodegeneration pathways. Gene Set Enrichment Analysis (GSEA) further revealed the upregulation of the apoptosis pathway and the downregulation of neuroactive ligand-receptor interaction. Protein–protein interaction (PPI) network analysis identified 10 hub genes, including COL18A1, CD63, and LTF. Immune infiltration analysis indicated that the occurrence of POD is strongly associated with immune cell activation, particularly in T cells and macrophages.

**Conclusion:**

Overall, this research primarily examines the intricate interplay between cell death processes and alterations in the immune microenvironment throughout the development of geriatric POD, pinpointing essential genes that provide vital theoretical support for further studies on geriatric POD. However, this discovery is only an initial one derived from analyzing the datasets. Upcoming research ought to evaluate and scrutinize additional datasets and conduct essential experiments to guarantee the precision and widespread relevance of the analytical findings.

## 1 Introduction

Postoperative delirium (POD) is a common perioperative neurological complication in the elderly, characterized by acute neurocognitive impairment that occurs over a short period (1). As society ages, the adverse effects of POD on the quality of postoperative recovery, healthcare costs, and mortality rates are increasingly being recognized. Even with the growing focus on preventing and treating POD, along with extensive experimental and clinical studies (2, 3), the fundamental molecular mechanisms of its pathogenesis remain mostly a mystery, highlighting the need for further research to improve treatment outcomes.

With the emergence of bioinformatics technologies, such as high-throughput sequencing, transcriptome analysis, and weighted gene co-expression network analysis (WGCNA) (4), the field of pathophysiological research on POD has significantly expanded, providing more precise directions for research. Current theories suggest that different types of programmed cell death could intensify neuroinflammation and oxidative stress, thereby accelerating the onset of POD (5, 6). Additionally, immune dysfunction is also thought to be closely associated with the occurrence of POD. These research findings offer new perspectives—integrating transcriptome data with immune characteristic analysis may help us gain a deeper understanding of the interactions and effects between neuroinflammation and immune responses in the development of POD (7, 8).

Our aim is to elucidate the precise link between POD and the diverse patterns of cell death using advanced analytical techniques, such as GSVA and WGCNA. The study also employed several methods, including differential expression analysis, functional enrichment analysis (9), and protein–protein interaction (PPI) network analysis (10, 11), to identify the pathogenic molecular pathways and key genes associated with the development of POD. Finally, we performed immune infiltration analysis to explore the roles of various immune cells in the progression of POD. By employing various bioinformatics methods, our comprehensive molecular analysis of POD in the elderly led to the discovery of its key pathogenic genes and novel therapeutic targets, establishing a solid foundation for future studies and the development of more effective diagnostic and treatment approaches.

## 2 Materials and methods

### 2.1 Data acquisition and preprocessing

Gene expression data were obtained from the GSE163943 dataset in the Gene Expression Omnibus (GEO) database. This dataset includes peripheral blood samples from four elderly patients (aged > 75) who developed POD after orthopedic surgery, as well as four age- and sex-matched non-POD orthopedic surgery patients. The original study employed a rigorous case–control matching design to ensure that there were no statistically significant differences in baseline characteristics between the two groups, including age, sex, body mass index (BMI), surgery duration, coronary heart disease (CHD), cerebrovascular disease (CVD), hypertension, and diabetes (all *p* > 0.05). All sample collections followed standardized preoperative fasting, anesthesia protocols, and postoperative care standards to minimize confounding factors. Ultimately, a total of 18,894 protein-coding genes were extracted for further analysis.

The key regulatory genes for 14 types of programmed cell death (PCD) patterns come from various sources, including the KEGG database (12), GeneCards database (13), Molecular Characterization database, Reactome database (14), and review articles (15, 16). The final gene list for the 14 different PCD patterns is provided in Supplementary material 1. This includes genes related to various types of cell death pathways: alkaliptosis (17) (7 genes), apoptosis (18) (136 genes), autophagy (19) (151 genes), cuproptosis (20) (14 genes), disulfidptosis (21) (4 genes), entotic cell death (22, 23) (15 genes), ferroptosis (24) (64 genes), lysosome-dependent cell death (255 genes), necroptosis (25) (27 genes), netotic cell death (26) (17 genes), oxeiptosis (27, 28) (26 genes), parthanatos (29–31) (9 genes), pyroptosis (32, 33) (27 genes), and lactylation (34) (333 genes). A total of 1,216 PCD-related genes were collected.

### 2.2 GSVA and cell death pathway analysis

Investigating the link between POD and different types of cell death, GSVA was performed using predefined gene sets corresponding to various cell death mechanisms. A heatmap was generated to visualize the correlation between POD occurrence and the types of cell death.

### 2.3 WGCNA

A gene co-expression network was constructed using the WGCNA package in R. Sample hierarchical clustering was performed to assess clustering quality and detect potential outliers. The soft-threshold power was determined using the “sft$powerEstimate” function to ensure a scale-free network topology. To distinguish unique gene modules, a baseline of 30 units was established. An analysis of module eigengene (ME) correlations was performed to explore the link between gene modules and the incidence of POD.

### 2.4 Differential expression analysis

DEGs between POD and normal samples were identified using the “limma” package in R (35). The thresholds were set to |log2(fold-change)| > 1 and *p* < 0.05.

### 2.5 Functional enrichment analysis

GO and KEGG pathway enrichment analyses were performed using the “clusterProfiler” package in R (36). GO enrichment concentrated on biological processes, molecular functions, and cellular components, whereas KEGG analysis identified key signaling pathways linked to POD. Additionally, GSEA was conducted to determine upregulated and downregulated pathways with statistical significance.

### 2.6 PPI network construction

Genes overlapping between DEGs and key WGCNA modules were imported into the STRING database (37) to construct a PPI network. Cytoscape software was used for network visualization and analysis (38). The MCC algorithm in the CytoHubba plugin was applied to identify the top 10 hub genes.

### 2.7 Immune infiltration analysis

The CIBERSORT (39) and XCELL (40) algorithms were used to evaluate immune cell infiltration levels in both POD and normal samples. The study examined the relationships between the top 10 hub genes and immune cell populations. Furthermore, a correlation analysis between immune-related genes and the hub genes was performed to explore their potential roles in immune regulation.

### 2.8 Statistical analysis

All statistical analyses were performed using R software (version 4.2.2). Pearson correlation analysis was used to assess associations between gene modules and POD. A *p*-value < 0.05 was considered statistically significant in all analyses.

## 3 Results

### 3.1 GSVA analysis

**Figure 1:**
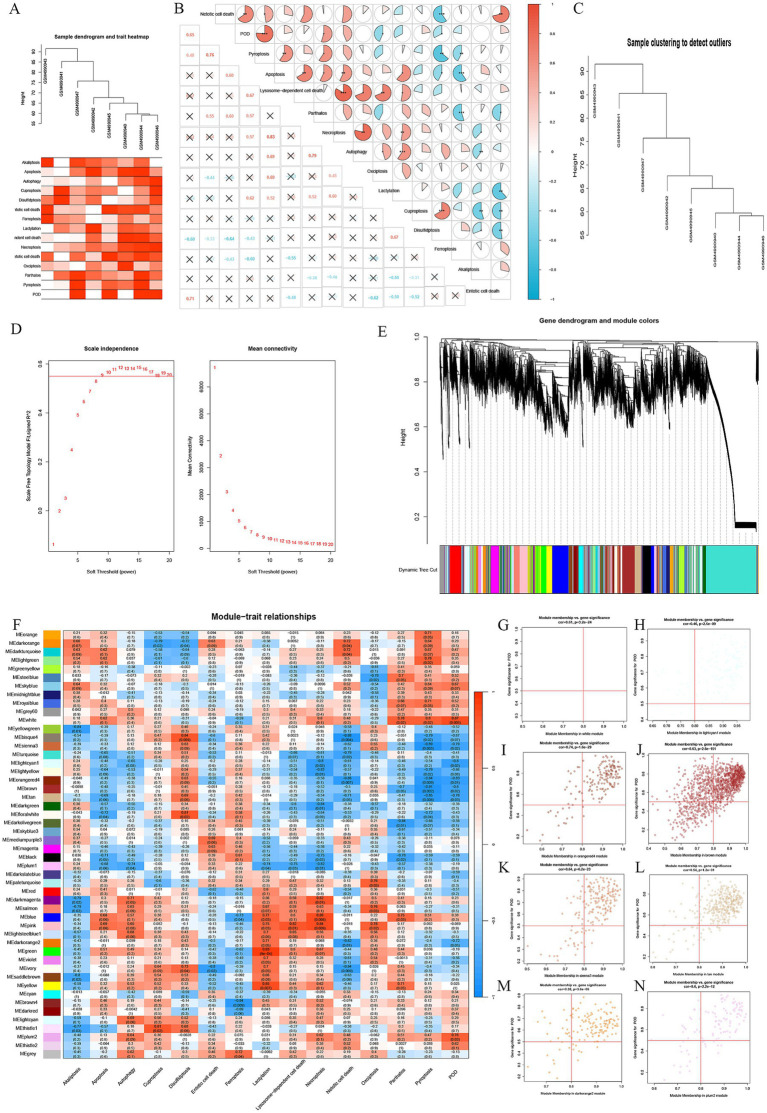
Association between POD and Cell Death pathways. **(A)** Heatmap of different types of cell death scores. **(B)** A heatmap depicts the relationship between various types of cell death and POD, with red indicating a positive correlation, blue indicating a negative one, and the completeness of the pie chart representing the strength of the correlation. * represents *p* < 0.05, ** represents *p* < 0.01, *** represents *p* < 0.001. **(C)** Sample dispersion. **(D)** WGCNA soft threshold. **(E)** Gene dendrogram and module colors. **(F)** A heatmap illustrating the relationship between different gene modules from WGCNA and different types of cell death, where red represents positive correlation and blue represents negative correlation. **(G–N)** Scatter plots demonstrated strong correlations between the GS and MM within the identified modules.

For evaluating the association between different types of cell death and POD in elderly patients, we first performed GSVA scoring and generated a heatmap that shows the relationship between the occurrence of POD and various types of cell death (Figure 1A). A correlation analysis between POD and different types of cell death in the dataset revealed that POD occurrence was positively correlated with Pyroptosis and Parthanatos, and negatively correlated with Oxidative Stress and Disulfidptosis, with statistical significance (Figure 1B).

### 3.2 WGCNA network construction and module analysis

Analyzing the raw data statistically reveals no notable disparities in clinical factors like age, BMI, duration of surgery, and comorbidities between the POD and non-POD groups. Based on the balanced baseline characteristics of both groups, we constructed a gene co-expression network in the GSE163943 dataset using the WGCNA algorithm. Sample hierarchical clustering analysis revealed strong clustering among the eight samples, with no obvious outliers (Figure 1C). The soft threshold power was established to be 9 using the “sft$powerEstimate” function (Figure 1D).

Gene hierarchical clustering dendrograms were constructed based on gene correlations, with a minimum module size of 30, identifying 50 distinct gene modules. A dendrogram was generated based on the dissimilarity measurement (1-TOM) for all genes (Figure 1E). The MEwhite module displayed a strong positive correlation with POD (*r* = 0.87, *p* = 0.005), as well as a strong positive association with Pyroptosis and Parthanatos. The MEsienna3 module showed a strong negative correlation with POD (*r* = −0.79, *p* = 0.02) and a strong negative correlation with Pyroptosis. The MElightcyan1 module exhibited a strong negative correlation with POD (*r* = −0.80, *p* = 0.02) and with Necroptosis. The MEorangered4 module was strongly negatively correlated with POD (*r* = −0.83, *p* = 0.01) and with Pyroptosis and Neotic cell death. The MEbrown module showed a strong negative correlation with POD (*r* = −0.80, *p* = 0.02) and with Pyroptosis. The MEtan module showed a strong negative correlation with POD (*r* = −0.76, *p* = 0.03) and with Pyroptosis and Neotic cell death. The MEdarkorange2 module displayed a strong negative correlation with POD (*r* = −0.72, *p* = 0.05), a strong negative correlation with Neotic cell death, and a strong positive correlation with Lactylation. Finally, the MEplum2 module showed a strong positive correlation with POD (*r* = 0.76, *p* = 0.03).

By integrating WGCNA modules with GSVA scores, a heatmap was created to visualize the correlations between different modules and types of cell death (Figure 1F). Scatter plots (Figures 1G–N) demonstrate strong correlations between Gene Significance (GS) and Module Membership (MM) within the identified modules, with all *p*-values being statistically significant (*p* < 0.05).

### 3.3 Differential expression analysis and module intersections

**Figure 2:**
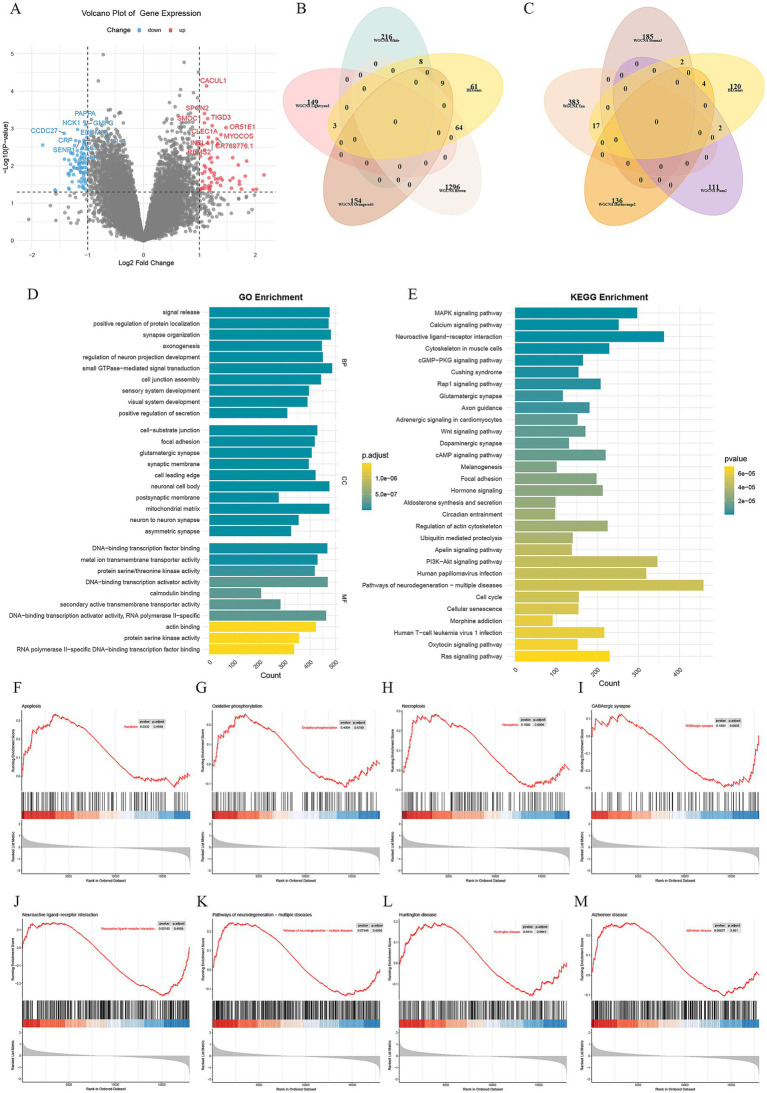
Differential expression analysis and functional enrichment analysis. **(A)** Volcano plot of differential analysis, where red represents upregulation and blue represents downregulation. **(B)** Modules with |r| ≥ 0.8 overlapped with DEGs. **(C)** Modules with |*r*| ≥ 0.7 overlapped with DEGs. **(D)** GO enrichment analysis. **(E)** KEGG enrichment analysis. **(F–M)** GSEA enrichment analysis.

The differential expression study of GSE163943 was performed with thresholds of |log2(fold-change)| > 1 and *p* < 0.05, identifying 145 DEGs, comprising 83 downregulated and 62 upregulated genes (Figure 2A). Modules with |r| ≥ 0.8 overlapped with DEGs, identifying common genes across MEwhite (8 genes), MElightcyan1 (3 genes), MEorangered4 (9 genes), and MEbrown (64 genes) (Figure 2B). Similarly, modules with |r| ≥ 0.7 identified common genes across MEsienna3 (2 genes), MEtan (17 genes), MEdarkorange2 (4 genes), and MEplum2 (2 genes) (Figure 2C).

### 3.4 Functional enrichment analysis

Enrichment analysis of the 145 DEGs showed that the GO terms were enriched in processes like regulation of neuron projection development, axonogenesis, synapse organization, neuron to neuron synapse, and glutamatergic synapse (Figure 2D). Analysis of the KEGG pathway revealed enrichment in pathways such as neuroactive ligand-receptor interaction, pathways of neurodegeneration (multiple diseases), calcium signaling pathway, PI3K-Akt signaling pathway, MAPK signaling pathway, circadian entrainment, and regulation of the actin cytoskeleton (Figure 2E). Furthermore, the KEGG analysis revealed enrichment in the GABAergic synapse pathway (Supplementary material 2).

GSEA indicated the upregulation of the apoptosis pathway and the downregulation of the neuroactive ligand-receptor interaction pathway, each showing statistical significance (Figures 2F,J). Additionally, there was a noticeable increase in oxidative phosphorylation, necroptosis, pathways of neurodegeneration (multiple diseases), Huntington disease, and Alzheimer disease pathways, while the GABAergic synapse pathway showed downregulation (Figures 2G–I,K–M).

### 3.5 PPI network analysis

**Figure 3:**
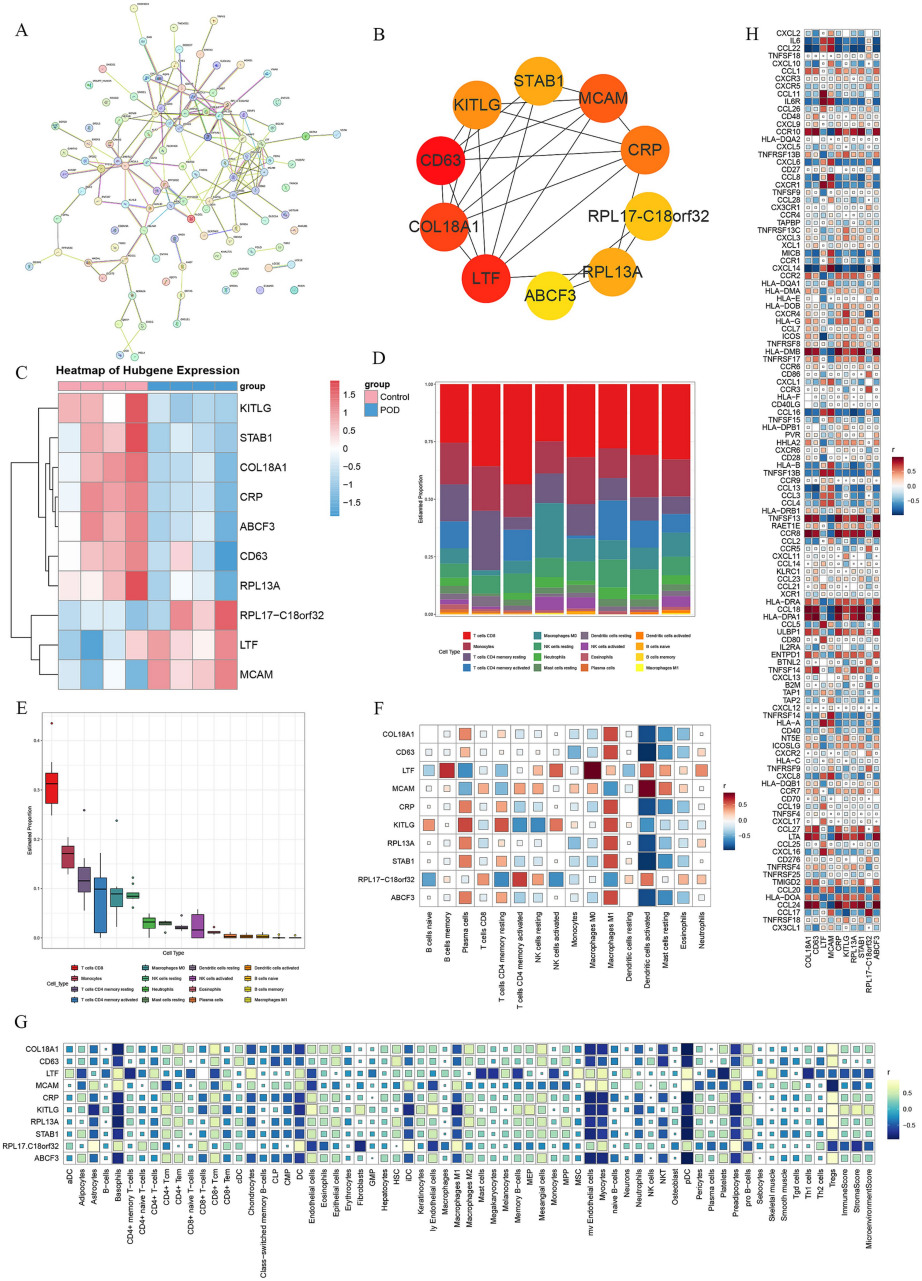
PPI network analysis and correlation between key genes and immune microenvironment. **(A)** PPI network diagram exported from the STRING website. **(B)** The top 10 genes were extracted using the MCC algorithm in Cytoscape software. **(C)** The heatmap displays the expression levels of the top 10 genes, with red indicating high expression and blue low expression. **(D)** CIBERSORT immune infiltration percentage bar plot. **(E)** CIBERSORT immune infiltration box plot. **(F)** The heatmap of the correlation between the top 10 genes and CIBERSORT immune infiltration, where red represents positive correlation and blue represents negative correlation. **(G)** A heatmap depicts the relationship between the leading 10 genes and xCell immune infiltration, with yellow-green indicating a positive link and cyan a negative one. **(H)** The heatmap of the correlation between the top 10 genes and 122 immune targets, where red represents positive correlation and blue represents negative correlation.

The genes from the intersecting modules with the DEGs were combined, culminating in a total of 109 genes. Subsequently, these genes were integrated into the STRING website to construct a PPI network (Figure 3A). Results from the PPI (Supplementary material 3) were transferred to Cytoscape software, where the MCC algorithm was used to extract the top 10 genes. These genes were COL18A1, CD63, LTF, MCAM, CRP, KITLG, RPL13A, STAB1, RPL17-C18orf32, and ABCF3 (Figure 3B). A heatmap of the top 10 genes in the expression profile was generated, showing that RPL17-C18orf32, LTF, and MCAM were upregulated in the POD samples, while COL18A1, CD63, CRP, KITLG, RPL13A, STAB1, and ABCF3 were downregulated in the POD samples (Figure 3C).

### 3.6 Correlation between key genes and immune microenvironment

Investigating the relationship between the top 10 genes and the immune microenvironment involved conducting further analysis of immune infiltration. CIBERSORT results revealed that CD8 T cells, Monocytes, memory CD4 T cells resting, memory CD4 T cells activated, and M0 Macrophages had higher proportions in the samples (Figures 3D,E). In the correlation between the top 10 genes and CIBERSORT results, Plasma cells, M1 Macrophages, Dendritic cells activated, and Mast cells resting were closely related. RPL17-C18orf32, LTF, and MCAM showed a negative correlation with Plasma cells and M1 Macrophages, but a positive correlation with Dendritic cells activated and Mast cells resting. COL18A1, CD63, CRP, KITLG, RPL13A, STAB1, and ABCF3 demonstrated a positive correlation with Plasma cells and M1 Macrophages, but a negative correlation with Dendritic cells activated and Mast cells resting. Additionally, LTF, KITLG, and RPL17 − C18orf32 exhibited a strong correlation with various immune cells (Figure 3F).

The immune infiltration analysis of the samples using XCELL showed a significant correlation between the top 10 genes and the XCELL results. The following cell types were closely related: Astrocytes, Basophils, CD4 + Tcm, CD4 + Tem, CD8 + Tcm, CD8 + Tem, Chondrocytes, Dendritic cells (DC), Endothelial cells, immature Dendritic cells (iDC), lymphatic Endothelial cells (ly Endothelial cells), Macrophages M2, microvascular Endothelial cells (mv Endothelial cells), Myocytes, Neutrophils, plasmacytoid Dendritic cells (pDC), Platelets, Preadipocytes, pro B-cells, and Tregs. RPL17-C18orf32, LTF, and MCAM showed a positive correlation with Astrocytes, Basophils, CD8 + Tem, Chondrocytes, DC, iDC, mv Endothelial cells, Myocytes, Neutrophils, pDC, Preadipocytes, and a negative correlation with CD8 + Tcm, Endothelial cells, ly Endothelial cells, Platelets, and Tregs. COL18A1, CD63, CRP, KITLG, RPL13A, STAB1, and ABCF3 exhibited a negative correlation with Astrocytes, Basophils, CD8 + Tem, Chondrocytes, DC, iDC, mv Endothelial cells, Myocytes, Neutrophils, pDC, Preadipocytes, and a positive correlation with CD8 + Tcm, Endothelial cells, ly Endothelial cells, Platelets, and Tregs (Figure 3G).

For a deeper exploration of the relationship between the top 10 genes and immune targets, a correlation study was conducted between the 122 immune-related targets (Supplementary material 4) and the top 10 genes. A strong link was observed between several cytokines and chemokines, immune receptors and ligands, antigen presentation, major histocompatibility complex (MHC)-related genes, ligands, and activation molecules with the top 10 genes. Specifically, RPL17-C18orf32, LTF, and MCAM demonstrated a positive correlation with multiple cytokines and chemokines, while exhibiting a negative correlation with several MHC-related genes. Conversely, COL18A1, CD63, CRP, KITLG, RPL13A, STAB1, and ABCF3 exhibited an inverse relationship with multiple cytokines and chemokines, while displaying a positive correlation with several MHC-related genes (Figure 3H).

## 4 Discussion

POD significantly increases the risk of postoperative complications and mortality in elderly patients, exerting considerable strain on healthcare resources for both families and society (1, 41). However, there is still a lack of effective pharmacological treatments for POD. Consequently, an extensive study and assessment were carried out to explore the link between 14 distinct forms of programmed cell death (PCD) (17–34) and the emergence of POD in elderly patients, with the aim of discovering better treatments. Through the application of protein–protein interaction (PPI) network technology, we effectively pinpointed crucial genes implicated in POD development and their effects on the immune microenvironment, thereby establishing a robust theoretical foundation for seeking more effective POD treatment approaches.

Each neuron requires a rich supply of blood oxygen, a stable immune system, and a balanced endocrine environment to ensure the normal transmission of neuronal signals and neurotransmitters, thus preserving brain function (42). However, in the perioperative phase, stressors such as surgical trauma, ischemia, or infection can modify the activity of crucial genes, potentially affecting the function of plasma cells, M1 macrophages, activated dendritic cells, and quiescent mast cells. This exacerbates the release of pro-inflammatory factors, such as IL-1β, IL-6, and TNF-*α*, which in turn regulate neuroinflammation, the balance of the blood–brain barrier (BBB), and imbalances in cellular metabolism by impacting pathways such as calcium, PI3K-Akt, and MAPK signaling, ultimately triggering POD in the elderly population (43, 44).

After screening, we identified 10 core genes closely associated with POD pathological changes, including RPL17-C18orf32, LTF, MCAM, COL18A1, CD63, CRP, KITLG, RPL13A, STAB1, and ABCF3. Within the POD specimens, there was a diminished expression of Col18a1, an essential element of the basement membrane. This decrease may be triggered by various mechanisms: firstly, diminished COL18A1 levels might interfere with the anchoring of proteins at endothelial cell junctions (like ZO-1 (45)), weakening the blood–brain barrier, thereby permitting external pro-inflammatory elements (such as IL-6, TNF-α) to infiltrate the central nervous system, intensifying inflammation in neural cells (46, 47); secondly, Col18A1 is responsible for coding the endostatin precursor protein, and its C-terminal non-collagenous domain (NC1) can emit endostatin with anti-angiogenic effects post-protease hydrolysis (48, 49). The downregulation of Col18A1 leads to a reduction in the levels of endothelial cell inhibitors, which in turn lessens the inhibition of MMP-9 activity (50) and promotes pathological restructuring of cerebral blood vessels (46, 51), thus intensifying ischemic harm in specific functional regions and brain malfunctions due to BBB permeability. In addition, Col18A1 reduces the binding region of its laminin, weakening the interaction between neurons and glial cells, which may result in neuronal dysfunction and a reduced ability to repair (52). Furthermore, some studies suggest that Col18A1 may affect cerebral blood supply by adjusting the release of neuroactive ligands or the sensitivity of receptors (53). These various changes act together, disrupting the protective function of the BBB, exacerbating neuroinflammation, and leading to the occurrence of POD.

The reduced expression of CD63 (lysosome-associated membrane protein) could indicate irregular immune interactions facilitated by extracellular vesicles (EVs). CD63-positive EVs typically carry anti-inflammatory molecules such as miR-124 (54, 55), capable of preventing the activation of pro-inflammatory M1-type microglia and A1-type astrocytes, thus safeguarding neurons by curbing neuroinflammation. Dysfunction of the GABAergic system is considered a key factor in the onset of delirium (56). A reduction in CD63 expression impacts lysosomal activity, potentially causing irregular regeneration of GABAergic synaptic vesicles and exacerbating the dysfunction of GABAergic synapses. Imbalance between excitatory neurotransmitters (such as glutamate) and inhibitory neurotransmitters (such as *γ*-aminobutyric acid, GABA) may have a negative impact on the consciousness level of elderly patients post-surgery. Given that benzodiazepines are widely and frequently used during the perioperative period for sedation and anesthetic induction, these medications enhance GABA_A receptors function and increase the efficacy of inhibitory neurotransmission (57). The interaction of these two elements can disturb synaptic balance, resulting in alterations in neural adaptability, which may potentially exacerbate cognitive impairments (58).

Neurons exhibit a high sensitivity to oxidative damage. The removal of RPL13A hinders the production of antioxidant enzymes (such as SOD), impacts the Nrf2/ARE pathway, worsens mitochondrial dysfunction, and elevates ROS levels (59, 60). An overabundance of peroxides triggers pathways like NF-κB, leading to the production of numerous inflammatory factors. The culmination of these reactions creates a harmful loop of inflammation and oxidative stress, which, in turn, impairs the synaptic plasticity of hippocampal neurons, negatively impacting cognitive abilities. The simultaneous downregulation of RPL13A and ABCF3 suggests a synergistic disruption of protein synthesis and metabolic homeostasis. A decline in RPL13A could result in issues with synaptic protein synthesis (61), and a decrease in ABCF3 expression might hinder the repair of damaged nerve cells, thus intensifying neurodegenerative changes.

It’s important to highlight that our research revealed an inverse relationship between oxidative stress and POD, with an increase in LTF observed in POD samples. This is in opposition to the findings of numerous studies, which suggest that oxidative stress contributes to delirium (62, 63). The following processes may be responsible for this variance: (1) The biphasic effect of oxidative stress: Moderate reactive oxygen species (ROS) can enhance cellular antioxidant defenses by activating the Nrf2/ARE pathway, whereas excessive ROS may lead to neuronal damage (64, 65). (2) Time-dependent effects: The data we gathered for our study relied on transcriptomic analysis of peripheral blood samples taken within 24 h after surgery, potentially revealing the physiological signaling roles of ROS in the initial stress stage (like aiding in tissue healing), as opposed to the long-term pathological consequences of accumulation. (3) In elderly patients, a decrease in fundamental antioxidant abilities (like lower glutathione levels) can lead to moderate oxidative stress, potentially enhancing neuroprotection through the Hormesis effect (an adaptive response triggered by small amounts of harmful substances) (66). (4) Our research focuses on patients aged 75 years or older who are undergoing orthopedic surgery. The distinct metabolic alterations they exhibit with age, such as defects in mitochondrial autophagy, could lead to varying levels of oxidative stress responses and roles compared to those in other groups, resulting in diverse outcomes (67).

Finally, we revealed the complex interaction between innate immunity and adaptive immunity in POD through immune microenvironment analysis. The positive correlation between COL18A1, CD63, plasma cells, and M1 macrophages may indicate an imbalance between anti-inflammatory reactions (like IL-10 release) (68, 69) and pro-inflammatory damage (like Tau phosphorylation) (70), potentially impacting cognitive abilities after surgery in older patients. Future research should incorporate multi-omics techniques (e.g., cerebrospinal fluid EVs miRNA sequencing) and gene knockout models under specific conditions (e.g., Cx3cr1-Cre mice) to explore the unique functions of these genes in BBB integrity, neuroimmune interference, and metabolic reprogramming, providing scientific evidence for targeted interventions.

Despite deriving numerous significant insights from our analysis, we must acknowledge certain limitations: First, the sample size of the dataset we used is relatively small (*n* = 8). Although the original study effectively controlled potential confounding factors such as age, gender, type of surgery, and comorbidities through a strict case–control matching design, the small sample size may lead to insufficient statistical power (such as the risk of false negatives), overfitting of gene expression variations, exaggerated false discovery rates, limited robustness in module detection, and reduced reliability of immune cell-related analysis results. Second, the analysis was limited to just one dataset (GSE163943), lacking experimental confirmation, potentially diminishing the precision and applicability of the findings and neglecting the intricate interconnections and characteristics of the data. Considering that there are relatively few datasets available for research related to POD in elderly patients, future studies might benefit from a second external group (like GSE174367) for comparative studies and additional clinical investigations. By enlarging the sample size and comparing it with cohorts from multiple centers, the precision and applicability of our current research findings can be further confirmed. Furthermore, the primary dataset collected for this research consists of peripheral blood samples. Although these blood samples are easier to obtain and the research findings can provide some guidance for POD treatment, the peripheral blood transcriptome may not fully capture the specific changes in the central nervous system. Therefore, future studies may need to incorporate cerebrospinal fluid proteomics or single-cell sequencing techniques to gain a deeper understanding of the mechanisms of neuroimmune interactions.

Even with some constraints, our research offers new data and guidance to support the advancement of treatment processes and the evolution of POD. With the aging population increasing, the effectiveness of POD in treating older patients continues to be inadequate. Therefore, it is critically important to persistently and thoroughly investigate this area to enhance postoperative recovery and improve the quality of life for older patients.

## 5 Conclusion

Overall, our research focused on 10 crucial genes closely linked to POD, exploring their roles in key signaling pathways, types of cell death, and changes in the immune microenvironment, with the aim of deepening our understanding of POD development in older individuals. Our study confirmed that the pathogenic origins of POD are complex in the elderly population, showing significant variation among patients. Therefore, future research should carefully assess these factors, integrating current findings with the unique circumstances of patients to develop more effective treatment strategies. This clarifies future research trends for POD in elderly patients and highlights the factors that need to be considered in subsequent experiments, thereby improving the applicability of the research findings and advancing clinical treatments.

## Data Availability

Gene expression data in our study were sourced from the GSE163943 dataset in the Gene Expression Omnibus (GEO) database. The Ethics statement for GSE163943 dataset can be accessed via this link: https://pmc.ncbi.nlm.nih.gov/articles/PMC8171121/#S7. Our study only analyzes the GSE163943 dataset and does not include any new animal or human research data.

## References

[1] Evered LA Chan MTV Han R Chu MHM Cheng BP Scott DA. Anaesthetic depth and delirium after major surgery: a randomised clinical trial. Br J Anaesth. (2021) 127:704–12. doi: 10.1016/j.bja.2021.07.021, PMID: 34465469 PMC8579421

[2] Wildes TS Mickle AM Ben Abdallah A Maybrier HR Oberhaus J Budelier TP. Effect of electroencephalography-guided anesthetic administration on postoperative delirium among older adults undergoing major surgery: the Engages randomized clinical trial. JAMA. (2019) 321:473–83. doi: 10.1001/jama.2018.22005, PMID: 30721296 PMC6439616

[3] Song Y Wang X Hou A Li H Lou J Liu Y. Integrative analysis of Lncrna and Mrna and profiles in postoperative delirium patients. Front Aging Neurosci. (2021) 13:665935. doi: 10.3389/fnagi.2021.665935, PMID: 34093168 PMC8171121

[4] Langfelder P Horvath S. Wgcna: an R package for weighted correlation network analysis. BMC Bioinformatics. (2008) 9:559. doi: 10.1186/1471-2105-9-559, PMID: 19114008 PMC2631488

[5] Xie Z Dong Y Maeda U Moir R Inouye SK Culley DJ. Isoflurane-induced apoptosis: a potential pathogenic link between delirium and dementia. J Gerontol A Biol Sci Med Sci. (2006) 61:1300–6. doi: 10.1093/gerona/61.12.1300, PMID: 17234824

[6] Wang P Velagapudi R Kong C Rodriguiz RM Wetsel WC Yang T. Neurovascular and immune mechanisms that regulate postoperative delirium superimposed on dementia. Alzheimers Dement. (2020) 16:734–49. doi: 10.1002/alz.12064, PMID: 32291962 PMC7317948

[7] Lu W Zhang K Chang X Yu X Bian J. The association between systemic immune-inflammation index and postoperative cognitive decline in elderly patients. Clin Interv Aging. (2022) 17:699–705. doi: 10.2147/CIA.S357319, PMID: 35535363 PMC9078355

[8] The Gene Ontology Consortium. The gene ontology resource: 20 years and still going strong. Nucleic Acids Res. (2019) 47:D330–8. doi: 10.1093/nar/gky1055, PMID: 30395331 PMC6323945

[9] Xu S Hu E Cai Y Xie Z Luo X Zhan L. Using Clusterprofiler to characterize multiomics data. Nat Protoc. (2024) 19:3292–320. doi: 10.1038/s41596-024-01020-z, PMID: 39019974

[10] Mosharaf MP Alam K Gow J Mahumud RA. Exploration of key drug target proteins highlighting their related regulatory molecules, functional pathways and drug candidates associated with delirium: evidence from Meta-data analyses. BMC Geriatr. (2023) 23:767. doi: 10.1186/s12877-023-04457-1, PMID: 37993790 PMC10666371

[11] van Wier SP Beekman AM. Peptide design to control protein-protein interactions. Chem Soc Rev. (2025) 54:1684–98. doi: 10.1039/D4CS00243A, PMID: 39817557 PMC11736853

[12] Kanehisa M Goto S Sato Y Furumichi M Tanabe M. Kegg for integration and interpretation of large-scale molecular data sets. Nucleic Acids Res. (2012) 40:D109–14. doi: 10.1093/nar/gkr988, PMID: 22080510 PMC3245020

[13] Liberzon A Birger C Thorvaldsdóttir H Ghandi M Mesirov JP Tamayo P. The molecular signatures database (Msigdb) Hallmark gene set collection. Cell Syst. (2015) 1:417–25. doi: 10.1016/j.cels.2015.12.004, PMID: 26771021 PMC4707969

[14] Milacic M Beavers D Conley P Gong C Gillespie M Griss J. The Reactome pathway knowledgebase 2024. Nucleic Acids Res. (2024) 52:D672–8. doi: 10.1093/nar/gkad1025, PMID: 37941124 PMC10767911

[15] Luo Y Liu L Zhang C. Identification and analysis of diverse cell death patterns in diabetic kidney disease using microarray-based transcriptome profiling and single-nucleus Rna sequencing. Comput Biol Med. (2024) 169:107780. doi: 10.1016/j.compbiomed.2023.107780, PMID: 38104515

[16] Zou Y Xie J Zheng S Liu W Tang Y Tian W. Leveraging diverse cell-death patterns to predict the prognosis and drug sensitivity of triple-negative breast Cancer patients after surgery. Int J Surg. (2022) 107:106936. doi: 10.1016/j.ijsu.2022.106936, PMID: 36341760

[17] Chen F Kang R Liu J Tang D. Mechanisms of Alkaliptosis. Front Cell Dev Biol. (2023) 11:1213995. doi: 10.3389/fcell.2023.1213995, PMID: 37601110 PMC10436304

[18] Perl M Chung C-S Ayala A. Apoptosis. Crit Care Med. (2005) 33:S526–9. doi: 10.1097/01.CCM.0000185499.28006.4C, PMID: 16340441

[19] Mizushima N Komatsu M. Autophagy: renovation of cells and tissues. Cell. (2011) 147:728–41. doi: 10.1016/j.cell.2011.10.026, PMID: 22078875

[20] Xie J Yang Y Gao Y He J. Cuproptosis: mechanisms and links with cancers. Mol Cancer. (2023) 22:46. doi: 10.1186/s12943-023-01732-y, PMID: 36882769 PMC9990368

[21] Liu X Zhuang L Gan B. Disulfidptosis: disulfide stress-induced cell death. Trends Cell Biol. (2024) 34:327–37. doi: 10.1016/j.tcb.2023.07.009, PMID: 37574347

[22] Krishna S Overholtzer M. Mechanisms and consequences of Entosis. Cell Mol Life Sci. (2016) 73:2379–86. doi: 10.1007/s00018-016-2207-0, PMID: 27048820 PMC4889469

[23] Florey O Kim SE Overholtzer M. Entosis: cell-in-cell formation that kills through entotic cell death. Curr Mol Med. (2015) 15:861–6. doi: 10.2174/1566524015666151026100042, PMID: 26511711

[24] Jiang X Stockwell BR Conrad M. Ferroptosis: mechanisms, biology and role in disease. Nat Rev Mol Cell Biol. (2021) 22:266–82. doi: 10.1038/s41580-020-00324-8, PMID: 33495651 PMC8142022

[25] Khoury MK Gupta K Franco SR Liu B. Necroptosis in the pathophysiology of disease. Am J Pathol. (2020) 190:272–85. doi: 10.1016/j.ajpath.2019.10.012, PMID: 31783008 PMC6983729

[26] Cai H Zeng Y Luo D Shao Y Liu M Wu J. Apoptosis and Netotic cell death affect diabetic nephropathy independently: an study integrative study encompassing bioinformatics, machine learning, and experimental validation. Genomics. (2024) 116:110879. doi: 10.1016/j.ygeno.2024.110879, PMID: 38851464

[27] Chen K-Q Wang S-Z Lei H-B Liu X. Mini-review: research and Progress of Oxeiptosis in diseases. Front Cell Dev Biol. (2024) 12:1428250. doi: 10.3389/fcell.2024.1428250, PMID: 38966429 PMC11222317

[28] Holze C Michaudel C Mackowiak C Haas DA Benda C Hubel P. Oxeiptosis, a Ros-induced caspase-independent apoptosis-like cell-death pathway. Nat Immunol. (2018) 19:130–40. doi: 10.1038/s41590-017-0013-y, PMID: 29255269 PMC5786482

[29] Fan F Yang C Piao E Shi J Zhang J. Mechanisms of chondrocyte regulated cell death in osteoarthritis: focus on Ros-triggered Ferroptosis, Parthanatos, and Oxeiptosis. Biochem Biophys Res Commun. (2024) 705:149733. doi: 10.1016/j.bbrc.2024.149733, PMID: 38442446

[30] Yang L Guttman L Dawson VL Dawson TM. Parthanatos: mechanisms, modulation, and therapeutic prospects in neurodegenerative disease and stroke. Biochem Pharmacol. (2024) 228:116174. doi: 10.1016/j.bcp.2024.116174, PMID: 38552851 PMC11410548

[31] Huang P Chen G Jin W Mao K Wan H He Y. Molecular mechanisms of Parthanatos and its role in diverse diseases. Int J Mol Sci. (2022) 23:7292. doi: 10.3390/ijms23137292, PMID: 35806303 PMC9266317

[32] Wei X Xie F Zhou X Wu Y Yan H Liu T. Role of Pyroptosis in inflammation and Cancer. Cell Mol Immunol. (2022) 19:971–92. doi: 10.1038/s41423-022-00905-x, PMID: 35970871 PMC9376585

[33] Rao Z Zhu Y Yang P Chen Z Xia Y Qiao C. Pyroptosis in inflammatory diseases and Cancer. Theranostics. (2022) 12:4310–29. doi: 10.7150/thno.71086, PMID: 35673561 PMC9169370

[34] Fan H Yang F Xiao Z Luo H Chen H Chen Z. Lactylation: novel epigenetic regulatory and therapeutic opportunities. Am J Physiol Endocrinol Metab. (2023) 324:E330–8. doi: 10.1152/ajpendo.00159.2022, PMID: 36856188

[35] Ritchie ME Phipson B Wu D Hu Y Law CW Shi W. Limma powers differential expression analyses for Rna-sequencing and microarray studies. Nucleic Acids Res. (2015) 43:e47. doi: 10.1093/nar/gkv007, PMID: 25605792 PMC4402510

[36] Wu T Hu E Xu S Chen M Guo P Dai Z. Clusterprofiler 4.0: a universal enrichment tool for interpreting omics data. Innovation. (2021) 2:100141. doi: 10.1016/j.xinn.2021.100141, PMID: 34557778 PMC8454663

[37] Szklarczyk D Kirsch R Koutrouli M Nastou K Mehryary F Hachilif R. The String database in 2023: protein-protein association networks and functional enrichment analyses for any sequenced genome of interest. Nucleic Acids Res. (2023) 51:D638–46. doi: 10.1093/nar/gkac1000, PMID: 36370105 PMC9825434

[38] Shannon P Markiel A Ozier O Baliga NS Wang JT Ramage D. Cytoscape: a software environment for integrated models of biomolecular interaction networks. Genome Res. (2003) 13:2498–504. doi: 10.1101/gr.1239303, PMID: 14597658 PMC403769

[39] Newman AM Liu CL Green MR Gentles AJ Feng W Xu Y. Robust enumeration of cell subsets from tissue expression profiles. Nat Methods. (2015) 12:453–7. doi: 10.1038/nmeth.3337, PMID: 25822800 PMC4739640

[40] Aran D Hu Z Butte AJ. Xcell: digitally portraying the tissue cellular heterogeneity landscape. Genome Biol. (2017) 18:220. doi: 10.1186/s13059-017-1349-1, PMID: 29141660 PMC5688663

[41] Swarbrick CJ Partridge JSL. Evidence-based strategies to reduce the incidence of postoperative delirium: a narrative review. Anaesthesia. (2022) 77:92–101. doi: 10.1111/anae.15607, PMID: 35001376

[42] Aksenov DP Gascoigne DA Duan J Drobyshevsky A. Function and development of interneurons involved in brain tissue oxygen regulation. Front Mol Neurosci. (2022) 15:1069496. doi: 10.3389/fnmol.2022.1069496, PMID: 36504684 PMC9729339

[43] Liu P Gao Q Guan L Hu Y Jiang J Gao T. Atorvastatin attenuates surgery-induced Bbb disruption and cognitive impairment partly by suppressing Nf-Κb pathway and Nlrp3 Inflammasome activation in aged mice. Acta Biochim Biophys Sin Shanghai. (2021) 53:528–37. doi: 10.1093/abbs/gmab022, PMID: 33674828

[44] Yang T Velagapudi R Terrando N. Neuroinflammation after surgery: from mechanisms to therapeutic targets. Nat Immunol. (2020) 21:1319–26. doi: 10.1038/s41590-020-00812-1, PMID: 33077953 PMC7704062

[45] Sasson E Anzi S Bell B Yakovian O Zorsky M Deutsch U. Nano-scale architecture of blood-brain barrier tight-junctions. eLife. (2021) 10:10. doi: 10.7554/eLife.63253, PMID: 34951586 PMC8747500

[46] Che J Sun Y Deng Y Zhang J. Blood-brain barrier disruption: a culprit of cognitive decline? Fluids Barriers CNS. (2024) 21:63. doi: 10.1186/s12987-024-00563-3, PMID: 39113115 PMC11305076

[47] Khoshneviszadeh M Henneicke S Pirici D Senthilnathan A Morton L Arndt P. Microvascular damage, Neuroinflammation and extracellular matrix remodeling in Col18a1 knockout mice as a model for early cerebral small vessel disease. Matrix Biol. (2024) 128:39–64. doi: 10.1016/j.matbio.2024.02.007, PMID: 38387749

[48] Brankin B Campbell M Canning P Gardiner TA Stitt AW. Endostatin modulates Vegf-mediated barrier dysfunction in the retinal microvascular endothelium. Exp Eye Res. (2005) 81:22–31. doi: 10.1016/j.exer.2005.01.005, PMID: 15978251

[49] Heljasvaara R Aikio M Ruotsalainen H Pihlajaniemi T. Collagen xviii in tissue homeostasis and dysregulation - lessons learned from model organisms and human patients. Matrix Biol. (2017) 57-58:55–75. doi: 10.1016/j.matbio.2016.10.002, PMID: 27746220

[50] Chaturvedi M Kaczmarek L. Mmp-9 inhibition: a therapeutic strategy in ischemic stroke. Mol Neurobiol. (2014) 49:563–73. doi: 10.1007/s12035-013-8538-z, PMID: 24026771 PMC3918117

[51] Simpson CE Griffiths M Yang J Nies MK Vaidya D Brandal S. Col18a1 genotypic associations with Endostatin levels and clinical features in pulmonary arterial hypertension: a quantitative trait association study. ERJ Open Res. (2022) 8:00725–2021. doi: 10.1183/23120541.00725-2021, PMID: 35769420 PMC9234438

[52] Guo S Wang H Yin Y. Microglia Polarization from M1 to M2 in neurodegenerative diseases. Front Aging Neurosci. (2022) 14:815347. doi: 10.3389/fnagi.2022.815347, PMID: 35250543 PMC8888930

[53] Wu Y-H Sun J Huang J-H Lu X-Y. Bioinformatics identification of angiogenesis-related biomarkers and therapeutic targets in cerebral ischemia-reperfusion. Sci Rep. (2024) 14:32096. doi: 10.1038/s41598-024-83783-9, PMID: 39738531 PMC11685884

[54] Fang Y Hong X. Mir-124-3p inhibits microglial secondary inflammation after basal ganglia hemorrhage by targeting Traf6 and repressing the activation of Nlrp3 Inflammasome. Front Neurol. (2021) 12:653321. doi: 10.3389/fneur.2021.653321, PMID: 34413820 PMC8369369

[55] Vaz AR Vizinha D Morais H Colaço AR Loch-Neckel G Barbosa M. Overexpression of Mir-124 in motor neurons plays a key role in Als pathological processes. Int J Mol Sci. (2021) 22:6128. doi: 10.3390/ijms22116128, PMID: 34200161 PMC8201298

[56] De Pace R Britt DJ Mercurio J Foster AM Djavaherian L Hoffmann V. Synaptic vesicle precursors and lysosomes are transported by different mechanisms in the axon of mammalian neurons. Cell Rep. (2020) 31:107775. doi: 10.1016/j.celrep.2020.107775, PMID: 32553155 PMC7478246

[57] Whiting PJ. Gaba-a receptor subtypes in the brain: a paradigm for Cns drug discovery? Drug Discov Today. (2003) 8:445–50. doi: 10.1016/S1359-6446(03)02703-X, PMID: 12801796

[58] Reimers A Odin P Ljung H. Drug-induced cognitive impairment. Drug Saf. (2024) 48:339–61. doi: 10.1007/s40264-024-01506-5, PMID: 39718691 PMC11903592

[59] Chauhan W Ferdowsi S Sudharshan SJ Zennadi R. Rpl13a Snornas-regulated Nadph oxidase 1-dependent Ros generation: a novel Rbc pathway mediating complement C3a deposition and triggering thrombosis in aging and venous blood clotting disorders. Free Radic Biol Med. (2025) 230:138–50. doi: 10.1016/j.freeradbiomed.2025.02.008, PMID: 39938620 PMC11936428

[60] Chauhan W Sj S Ferdowsi S Sohel A Zennadi R. Red blood cell Rpl13a small noncoding nucleolar RNAs guides 2’-O-methylation on peroxidasin messenger RNA promoting venous thrombosis in aging. J Thromb Haemost. (2025). 7:S1538-7836(25)00135-7. [ahead of print]. doi: 10.1016/j.jtha.2025.02.036, PMID: 40058703 PMC12353407

[61] Klein ME Monday H Jordan BA. Proteostasis and Rna binding proteins in synaptic plasticity and in the pathogenesis of neuropsychiatric disorders. Neural Plast. (2016) 2016:3857934–11. doi: 10.1155/2016/3857934 26904297 PMC4745388

[62] Yuba T Koyama Y Takahashi A Fujino Y Shimada S. Association between oxidative stress and postoperative delirium in joint replacement using Diacron-reactive oxygen metabolites and biological antioxidant potential tests. Sci Rep. (2024) 14:29854. doi: 10.1038/s41598-024-80739-x, PMID: 39617794 PMC11609295

[63] Karlidag R Unal S Sezer OH Bay Karabulut A Battaloğlu B But A. The role of oxidative stress in postoperative delirium. Gen Hosp Psychiatry. (2006) 28:418–23. doi: 10.1016/j.genhosppsych.2006.06.002 16950378

[64] Ngo V Duennwald ML. Nrf2 and oxidative stress: a general overview of mechanisms and implications in human disease. Antioxidants. (2022) 11:2345. doi: 10.3390/antiox11122345, PMID: 36552553 PMC9774434

[65] Yu T Ding C Peng J Liang G Tang Y Zhao J. Sirt7-mediated Nrf2 deacetylation promotes antioxidant response and protects against Chemodrug-induced liver injury. Cell Death Dis. (2025) 16:232. doi: 10.1038/s41419-025-07549-5, PMID: 40169535 PMC11961749

[66] Calabrese V Cornelius C Dinkova-Kostova AT Calabrese EJ Mattson MP. Cellular stress responses, the Hormesis paradigm, and Vitagenes: novel targets for therapeutic intervention in neurodegenerative disorders. Antioxid Redox Signal. (2010) 13:1763–811. doi: 10.1089/ars.2009.3074, PMID: 20446769 PMC2966482

[67] Tran M Reddy PH. Defective autophagy and Mitophagy in aging and Alzheimer's disease. Front Neurosci. (2020) 14:612757. doi: 10.3389/fnins.2020.612757, PMID: 33488352 PMC7820371

[68] Porro C Cianciulli A Panaro MA. The regulatory role of Il-10 in neurodegenerative diseases. Biomol Ther. (2020) 10:1017. doi: 10.3390/biom10071017, PMID: 32659950 PMC7407888

[69] Bido S Nannoni M Muggeo S Gambarè D Ruffini G Bellini E. Microglia-specific Il-10 gene delivery inhibits Neuroinflammation and neurodegeneration in a mouse model of Parkinson's disease. Sci Transl Med. (2024) 16:eadm8563. doi: 10.1126/scitranslmed.adm8563, PMID: 39167665

[70] Liu C Zhang C Chen L Liu X Wu J Sun Y. Lingo1 in the Hippocampus contributes to cognitive dysfunction after anesthesia and surgery in aged mice. Int J Biol Sci. (2025) 21:595–613. doi: 10.7150/ijbs.98376, PMID: 39781463 PMC11705636

